# Wild Chimpanzees Exchange Meat for Sex on a Long-Term Basis

**DOI:** 10.1371/journal.pone.0005116

**Published:** 2009-04-08

**Authors:** Cristina M. Gomes, Christophe Boesch

**Affiliations:** Max Planck Institute for Evolutionary Anthropology, Leipzig, Germany; Indiana University, United States of America

## Abstract

Humans and chimpanzees are unusual among primates in that they frequently perform group hunts of mammalian prey and share meat with conspecifics. Especially interesting are cases in which males give meat to unrelated females. The meat-for-sex hypothesis aims at explaining these cases by proposing that males and females exchange meat for sex, which would result in males increasing their mating success and females increasing their caloric intake without suffering the energetic costs and potential risk of injury related to hunting. Although chimpanzees have been shown to share meat extensively with females, there has not been much direct evidence in this species to support the meat-for-sex hypothesis. Here we show that female wild chimpanzees copulate more frequently with those males who, over a period of 22 months, share meat with them. We excluded other alternative hypotheses to exchanging meat for sex, by statistically controlling for rank of the male, age, rank and gregariousness of the female, association patterns of each male-female dyad and meat begging frequency of each female. Although males were more likely to share meat with estrous than anestrous females given their proportional representation in hunting parties, the relationship between mating success and sharing meat remained significant after excluding from the analysis sharing episodes with estrous females. These results strongly suggest that wild chimpanzees exchange meat for sex, and do so on a long-term basis. Similar studies on humans will determine if the direct nutritional benefits that women receive from hunters in foraging societies could also be driving the relationship between reproductive success and good hunting skills.

## Introduction

Evidence from studies on hunter-gatherer societies suggests that men who are more successful hunters have higher reproductive success [1]. One of the hypotheses proposed to explain this relationship is the meat-for-sex hypothesis, whereby men and women exchange meat for mating access [2]. Better hunters have been shown to have a higher mean number of extra-marital affairs [1]–[3], and in groups in which polygynous marriages occurred frequently, more productive hunters had more wives [3]. These findings have been attributed to direct family provisioning (i.e. kin selection, [3]), and to hunters being preferred mating partners due to their high social status, through which they might be signaling their good genes (i.e. costly signaling theory, [4], [5]). However, there is little evidence in humans supporting any of the mechanisms leading to the relationship between increased reproductive success and good hunting skills [1], [3]. Understanding female choice and male-female meat sharing in species in which copulation frequency can be easily measured and dyadic transfers of meat can be directly quantified is likely to give us insight into the mechanisms driving the latter relationship in humans.

Among chimpanzees, humans' closest living relative, male hunters also share meat with unrelated females [6]–[8]. The meat-for-sex hypothesis is a plausible explanation for male-female meat-sharing in this species [7], as chimpanzees are highly promiscuous [9]–[11], they have a certain degree of female choice [12], and hunters can usually control the sharing of their catch [6], [9]. Hunting and sharing meat in the Taï chimpanzee community was attributed to the nutritional benefits of consuming meat, and shown to be stabilized by mutualism, as hunters, which are usually males, attained the largest share of the hunt, and gained more by hunting together than by hunting alone [6], [13]. In other populations, males also ate more meat than females, and frequently shared meat and exchanged meat for other social services amongst each other (e.g. meat for support in agonistic conflicts, [14]). However, the mechanisms driving the transfers of meat from males to females, which occur less frequently, are still poorly understood. The meat-for-sex hypothesis has been proposed to explain these cases [6], [7], although support for this hypothesis in chimpanzees has been limited. Males in the Taï chimpanzee community frequently shared meat with females that did not contribute to the hunt, which could be potential cases of exchange of meat for sex [6]. Females in the Gombe community, on the other hand, needed to hunt to acquire comparable amounts of meat as males, since males rarely shared meat with females in this community [6]. In the Ngogo and Gombe chimpanzee communities, multivariate analyses showed that the probability of hunting did not increase with the number of estrous females (i.e. cycling females with sexual swellings [15]) present in the hunting party ([14], [16]–[18], see [19] for an exception), and males did not share more frequently with estrous females than with anestrous ones [14], [20]. This suggests that males were not diverting resources towards females with whom they could potentially copulate on a short-term, and were therefore not exchanging meat for sex. Although males in the Ngogo community did share more with estrous than anestrous females given their proportional representation in hunting parties [14], these sharing episodes did not confer males with immediate mating advantages over males who did not share meat with females (Ngogo: [14], Gombe: [20]). One important limitation of the above studies is that they have focused on short-term exchanges of meat for sex (i.e. within the estrous phase of the female). Given that in this species dyads form long-lasting bonds [21], and exchange services on a long-term basis [22], it is plausible that exchanges of meat for sex could occur over a longer period of time than the estrous phase of a female.

To test this hypothesis, we collected data on meat sharing and copulation frequency in a group of wild chimpanzees in the Taï National Park, Côte d'Ivoire between 2003 and 2006. At the time of data collection, the group consisted of 49 individuals, 5 adult males and 14 adult females. Eight of the 14 adult females were in estrous at some point during the study period. Because male chimpanzees only copulate with estrous females, the analysis was restricted to pairs formed by these females and the five adult males.

Meat sharing was scored whenever meat was transferred from one individual to another in an apparently voluntary fashion (cases of theft in which the male screamed, cried or aggressed against the female after the transfer, were excluded from the analysis). Transfers could be either passive (e.g. a male allowed a female to co-feed on a piece of meat he clearly owned) or active transfers (e.g. a male tore a limb from the carcass and placed it in the outstretched hand of a female, [23]), and we recorded both the frequency and amount of meat transferred in each case (see Methods).

## Results and Discussion

At least 1 estrous female was present at 64 of the 90 successful hunts observed (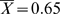 estrous females per hunt, First quartile (Q1) = 0, Third quartile (Q3) = 1), and at least 1 anestrous female was present at 81 successful hunts (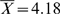 anestrous females per hunt, Q1 = 2.25, Q3 = 6). We recorded 262 male to female meat transfers. On average males shared with 5.6 of the 8 females that were in estrous during the data collection period (Q1 = 5, Q3 = 8). The number of times (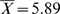, Q1 = 0, Q3 = 9) and the amount of meat (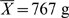, Q1 = 0 g, Q3 = 1356 g) that each male shared with each female over the entire study period varied considerably. These findings suggest that males shared unevenly with females, and therefore, the caloric benefits that each female gained from each male differed.

We observed a total of 262 copulations during the 1814 h that females were observed in estrous (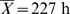 per female, Q1 = 211, Q3 = 265). Of the 39 adult male-estrous female dyads that were seen together during the estrous phase of the female, 30 were observed to copulate at least once. Females generally did not copulate with all of the adult males of the group (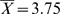 males per female, Q1 = 3, Q3 = 4.25), and there was considerable variation in the number of times they copulated with each one of their mating partners (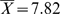 times per pair, Q1 = 2, Q3 = 11). These findings indicate that females copulated unequally with males, and therefore, males' mating success with each female varied. Finally, out of the 30 male-estrous female dyads that were observed copulating, in 9 dyads (30%) the male did not share meat with the female, while in 21 dyads (70%) the male did share meat with the female throughout the entire study period.

Generalized Linear Mixed Models (GLMM, [24]) with a Gaussian error structure were used to test the hypothesis that males and females exchange meat for mating opportunities. We set as the response variable the total number of copulations per male-female dyad, and as the binary predictor variable, whether or not the male had transferred meat to the respective female over the entire study period. This analysis allowed us to investigate whether females copulated more frequently with males who shared meat with them than with males who never shared meat with them, irrespective of the amount shared. We did two additional analyses in which the total number of sharing events was the predictor variable in one, and the amount of meat (in grams, g) shared by a male in each male-female dyad was the predictor variable in the other. These allowed us to investigate whether females copulated more frequently with those males who shared more frequently with them, or with those who shared more meat with them.

**Figure 1:**
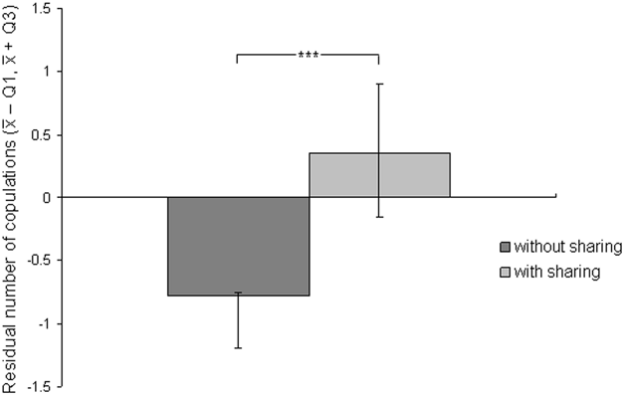
Average residual number of copulations for dyads in which the male did, and did not share meat with the female. The residuals were obtained from a model where number of copulations per dyad was the response variable and rank of the male, age of the female, DAI during the entire study period and during the estrous phase of the female, female gregariousness, and female begging were predictor variables. (*** indicates p<0.0001).

We found that females copulated more frequently with males who shared meat with them at least on one occasion, than with males who never shared meat with them (GLMM, estimate±se = 1.4±0.21, t_30_ = 6.43, p<0.0001), indicating that sharing meat with females improved a males' mating success. To investigate the possibility that female preference could also be influenced by other male-female positive social interactions, such as grooming, giving support in agonistic conflicts or sharing other non-meat food items, we additionally included these interactions in the statistical model (see Methods). Overall, the predictor variables included in the model had a significant effect on copulation frequency (likelihood-ratio test: χ^2^ = 27.64, df = 4, N = 30, p<0.0001). However, we found that in this model only sharing or not sharing meat with females was significant (GLMM, estimate±se = 1.75±0.28, t_30_ = 6.21, p<0.0001). Neither grooming (GLMM, estimate±se = 0.008±0.06, t_30_ = 0.15, p = 0.88), sharing other food items (GLMM, estimate±se = −0.1±0.26, t_30_ = −0.39, p = 0.69) or supporting a female in agonistic interactions (GLMM, estimate±se = 0.52±0.29, t_30_ = 1.79, p = 0.08) significantly affected a males' mating success, although the latter tended to influence it. These results indicate that meat sharing was the principal positive interaction between males and females that influenced a males' mating success, which suggests that males and females were exchanging meat for sex. However, a spurious relationship between mating success and sharing meat could arise if high-ranking males were both copulating and sharing meat more frequently than low-ranking ones. It could also result from males sharing meat and copulating more frequently with preferred, high-ranking or old females [25], or with those that frequently associated with them. The above relationship could also occur if females that were more gregarious, and in general associated more frequently with males, copulated more often and were more likely to receive meat from males. Finally, if a female that copulated frequently with a particular male was more likely to harass that male, and by doing so persuade the male to share meat with her and thus avoid the costs of harassment [20], [26], then a spurious relationship between meat sharing and copulation could arise. Although females in the Taï community rarely harassed males in the costly way described in other chimpanzee populations (i.e. prevented the male from eating by holding the carcass, or covering the males' mouth, [20]), they did frequently beg meat from them (e.g. extended their cupped hand towards the male, or cried next to the male). We excluded all the above alternative hypotheses by controlling statistically for rank of the male, rank or age of the female, the association patterns between males and females, both during the estrous phase of the female and throughout the whole data collection period, the level of gregariousness of the female, and the number of hunts in which each female begged meat from each male (see Methods section for details). Overall, the predictor variables included in this model had a significant effect on copulation frequency (likelihood-ratio test: χ^2^ = 32.83, df = 7, N = 30, p<0.0001). We found that after controlling for the effect of these other predictor variables, the relationship between meat-sharing and copulation frequency remained significant (Table 1). This indicates that females copulated more with males who shared meat with them than with males who did not share meat with them (Fig. 1), irrespective of the rank of the male, the rank or age of the female, the association patterns of the dyad, the level of gregariousness of the female and the begging tendencies of each female towards each male. A model which included all of the predictor variables except meat-sharing had a significantly inferior fit (Model with meat shared: AIC = 69, df = 11, N = 30; Model without meat shared: AIC = 89, df = 10, N = 30; likelihood-ratio test: χ^2^ = 21.51, p<0.0001), indicating that sharing meat explained an important amount of the variability of male mating success. The resulting equation of copulation frequency (copulation frequency (y) = intercept+estimate meat sharing*x: y = 1.55+1.56*x, see Table 1) indicates that if a male shared meat with a particular female, his mating success on average was twice as large (y = 1.55+1.56*1 = 3.11) as if he did not share meat with the female (y = 1.55+1.56*0 = 1.55). Our findings suggest that male and female wild chimpanzees exchange meat for sex, i.e. males increase their mating success by sharing meat with females, and females increase their caloric intake by copulating more frequently with those males who share meat with them.

**Table 1:** Factors influencing the number of times each male-female dyad copulated.

Predictor variable	b±se	t	p-value
Intercept	1.55±2.84	0.54	0.59
Sharing meat	1.56±0.33	4.69	0.0001
Male rank	0.21±0.09	2.19	*0.03*
Female age	0.03±0.02	1.34	0.19
Female gregariousness	−2.69±5.12	−0.53	0.60
Estrous DAI	0.02±0.78	0.03	0.97
Total DAI	2.46±3.57	0.69	0.49
Begging	−0.05±0.09	−0.60	0.55

Predictor variables: Sharing or not sharing meat with females over the entire study period (binary variable), rank of the male (which was stable throughout the whole period), age of the female (a separate model with rank of the female instead of age, revealed the same results regarding statistical significance of the predictor variables), gregariousness of the female, association patterns of the male and female (DAI), both throughout the entire study period (Total DAI) and during the estrous phase only (Estrous DAI), and number of hunts in which each female begged meat from each male.

Limiting the analysis to dyads that both shared meat and copulated indicated that there was no obvious linear relationship between mating success and sharing meat (GLMM_for amount of meat shared_, estimate±se = 0.0001±0.0002, t_21_ = 1.19, p = 0.25; GLMM_for frequency of sharing_, estimate±se = 0.003±0.03, t_21_ = 0.08, p = 0.93). This indicates that females did not copulate more frequently with males who shared more meat with them than with those who shared less meat. Thus, males managed to increase their mating success by sharing meat with females; however, the frequency and amount of meat they shared with each female did not affect their probability of mating more frequently. Rank of the male did have a linear relationship with mating success, indicating that high ranking males copulated more frequently than low ranking males (Table 1). Male rank and sharing meat with females had independent effects on male mating success, indicating that females copulated more with males who shared meat with them and with males of high rank.

**Figure 2:**
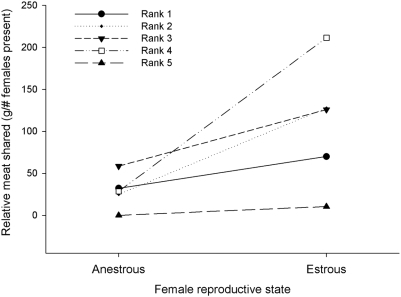
Standardized amount of meat each male shared with estrous and anestrous females. We used a standardized value of meat shared to control for the number of females present of the reproductive state of the female with whom the male shared meat. This allowed verification of whether males shared more meat with estrous than anestrous females given their proportional representation in hunting parties. A model with frequency of sharing instead of total amount revealed essentially the same results.

As in other chimpanzee populations [14], [20], males did not share meat more frequently, or in larger amounts, with estrous (49 times, 5.8 kg) than with anestrous females (213 times, 32.76 kg). However, this could be due to the presence of more anestrous than estrous females in hunting parties. To control for this possibility, we standardized the frequency/amount of meat shared on each occasion by dividing the said amount by the number of females of the same reproductive state as the female who received meat, present in the party when the transfer took place (see Methods). We found that males tended to share more with estrous than with anestrous females, given their proportional representation in hunting parties (Fig. 2; Wilcoxon exact signed-ranks test: T+ = 15, N = 5, p = 0.0625, this is the smallest p-value that an exact test with a sample size of five can reveal). These findings indicate that although males tended to preferentially share meat with females that were in estrous, they were not investing exclusively in them. This could suggest that the exchange of meat for sex did not occur only during the estrous phase of females (i.e. short-term), but rather throughout both of their reproductive periods (i.e. long-term). To test for this possibility, we excluded from the analysis meat transfers towards estrous females. In this analysis, the relationship between mating success and sharing or not sharing meat (binomial predictor variable) remained significant (GLMM: estimate±sd = 1.25±0.34, t_30_ = 3.58, p = 0.001). Furthermore, when evaluating the model only with the transfers done towards estrous females, the relationship between mating success and sharing or not sharing meat disappeared (GLMM: estimate±sd = 0.23±0.43, t_30_ = 0.54, p = 0.59). These results suggest that males and females exchanged meat for sex on a long-term basis. Although the latter result could be due to low power because males had fewer opportunities to share meat with estrous females, it indicates that short-term exchanges alone cannot account for the relationship between sharing meat and mating success.

Our findings show that females copulated more frequently with those males who shared meat with them over long periods of time. Previous studies in Taï showed that females were very successful at obtaining meat independently of their contribution during the hunt [6]. Although hunting in chimpanzees is likely to be driven by the nutritional and social benefits that males gain by sharing meat amongst each other [6], [14], [16], our present results suggest that cases of male to female meat transfer in the Taï chimpanzee community are best explained by the meat-for-sex hypothesis. Studies on other chimpanzee populations have shown that the presence of estrous females does not increase the probability of hunting more frequently (Ngogo: [14]) and in some cases even decreases hunting probability (Gombe: [16], [17]). These findings have been attributed to males facing a trade-off between mate-guarding estrous females so as to prevent other males from copulating with them, and leaving these females to hunt for meat [20]. However, this scenario does not preclude and in fact supports the possibility that if females prefer as mating partners, males who share meat with them, then these exchanges should take place on a long-term basis. We propose that in chimpanzee populations where female choice is present and hunters can usually control the sharing of their catch, male and female wild chimpanzees will exchange meat for sex over long periods of time. Previous studies might have not found a relationship between mating success and meat sharing because they focused on more short-term exchanges, or perhaps because in these groups female choice was rare (i.e. males coerced females [27]), hence, exchanging meat for sex was not a viable strategy. Our study shows that like human foragers, male chimpanzees can increase their mating success by hunting and sharing meat with females, while females can increase their meat intake by mating more frequently with males who share meat with them. Further studies on other chimpanzee communities which take into account the degree of female choice in the group and the capacity of hunters to control the sharing of their catch will determine whether these exchanges can be generalized to the species. Our findings also shed light on our current knowledge on meat sharing in humans [28]–[33], suggesting that the increased reproductive success of accomplished hunters compared to unsuccessful hunters in forager societies could be driven by female choice and be linked to direct exchanges of meat for sex between men and women. Future studies on human foragers, which adopt methods used by field ecologists, will allow the testing of this hypothesis.

## Methods

### Ethics statement

This research complied with the ethics guidelines of the Max Planck Society and was supported by the Ivorian authorities (Ivory Coast Park Authorities and Ministry of Science and Research).

### Details of the data collection of meat sharing events

Data on meat sharing were collected by C.M.G. with the help of two field assistants, and recorded into a handheld computer. We collected meat sharing data through *ad-libitum* observations of hunting behavior and meat eating parties, while data on copulations and other behaviors (e.g. grooming, support in agonistic conflicts, association patterns, rank, etc.) came from 3000 h of focal target follows [22]. Because the number of adults present in each meat eating party was relatively small (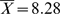 adults per hunt, Q1 = 6, Q3 = 10), and in most cases only one monkey was captured at a single time, it was possible, between the two to three observers, to track most of the primary meat transfers. The amount of meat transferred from one individual to another was estimated based on body-part weight (obtained from the literature, in cases when a complete body part was transferred, [34]–[36]), relation of meat transferred to chimpanzee hand size (in cases when single pieces were transferred; a 5×5 cm piece of tissue from a red colobus weighed 50 g, K. Mätz-Rensing pers. comm.) and/or time spent eating another individuals' piece (in cases when individuals were allowed to eat from pieces that others clearly monopolized; estimated by calculating the proportion of the meat portion shared, that the time each individual spent feeding on it represented).

### Statistical analysis and model specifications

General results were presented using the trimmed mean which is sensitive to skewed data [37], the first quartile (Q1) and third quartile (Q3) throughout.

To study the relationship between mating success and meat sharing we used GLMM [24] and set as the response variable the dyadic copulation frequency of each male-female dyad and as a predictor variable male-female meat sharing. Since male chimpanzees copulate with females throughout their entire estrous phase, the analysis presented here includes copulations that females had both during their maximal swelling period and partial swelling periods [15]. However, including only copulations that took place during females' maximal swelling phase did not significantly change the results. We ran three separate analyses using different measures of meat sharing in each one: (1) If a male shared meat with a female at least on one occasion throughout the entire study period (binary variable); (2) The total number of times a male shared meat with a female throughout the entire study period; and (3) The total amount of meat (g) a male shared with a female throughout the entire study period. With each of these measures of meat sharing we ran two different models with a different set of additional predictor variables. We did this to test alternative explanations to having a relationship between meat sharing and copulation frequency and additionally to reduce, in each case, the number of predictor variables included in the model. In the first model we included as predictor variables, positive behaviors that a male can direct towards a female: (a) the amount of time each male groomed each female (sec), and whether or not each male (b) shared meat, (c) supported or (d) shared other non-meat food items (e.g. *Treculia* fruit, nuts, or other divisible foods) with each female. In the second model, in addition to meat sharing, we included all variables that have or might have an effect on male mating success, female choice or meat sharing: (a) Rank of the male and female, determined based on greeting vocalizations [38]; (b) Age of the female (because female rank and age were correlated: r^2^ = 0.63, N = 9, p = 0.01, we ran two separate models, one using rank and the other age, and obtained the same results regarding the statistical significance of each predictor variable); (c) Association patterns of each male-female dyad both during the entire study period, and during the estrous phase of the female, based on the Dyadic Association Index (DAI, [22], [39]), (d) Gregariousness of the female, based on the amount of time each female target was seen accompanied by at least one other adult chimpanzee; and (e) Female begging, based on the number of hunts in which each female begged from each male. In addition to the above predictor variables set as fixed effects, the identity of the male and female were set as random effects in all models. This relieves the problem of dependency of data by controlling for the variation among individuals in their tendency to perform certain acts (e.g. sharing meat or copulating, [40]). To investigate whether the time we observed each male-estrous female dyad or each male-female dyad together in hunting parties affected the results we obtained, we ran a model using as the response variable copulations per unit time that the dyad was observed, and as a predictor variable the amount of meat shared per dyad in relation to the number of hunts in which the male-female dyad was present. We obtained the same results, regarding the statistical significance of the predictor variables, as for the analysis using total amounts.

To investigate whether males shared more meat with estrous than with anestrous females given their proportional representation in hunting parties, we controlled for the number of females of the reproductive state of the female who received meat, present in the hunting party at the time of the transfer. We did this by calculating the standardized amount of meat a male shared with a female and did a Wilcoxon exact signed-ranks test. The standardized amount of meat that a male shared was obtained the following way: for each sharing event, we divided the amount of meat the male shared with the female by the number of potential sharing partners, of the same reproductive state as the female with whom the male shared, available to the male (e.g. if a male shared with an estrous female, we divided the amount of meat shared by the number of estrous females present). We then added all the sharing events of each male-female dyad, separately for estrous and anestrous females, and averaged the obtained values per male. A model with frequency of sharing instead of total amount revealed the same results regarding the statistical significance of the predictor variables.

To assess the overall significance of each model, we compared it to the null model (i.e. one that only included the intercept and the random variables) by performing a likelihood-ratio test that compared the log-likelihoods of both. To investigate whether the model was unstable due to multicollinearity between two or more predictor variables, the data were bootstrapped 1000 times to obtain parameter coefficients of each of the individual predictor variables [41]. This allowed us to verify that the confidence intervals (CI) for parameter estimates of significant variables were small and did not include zeros, which is evidence for a minor effect of multicollinearity. Because measures of effect size are not available for GLMM, we evaluated effect size by comparing the AIC of the full model and a reduced model which did not include the variable of interest and performed a likelihood-ratio test. This allowed us to assess the amount of variability explained by a single variable [42]. All analyses were carried out in R [43], using for GLMM the lme4 package version 0.9975–13 [44].
